# Alternative therapeutics for self-limiting infections—An indirect approach to the antibiotic resistance challenge

**DOI:** 10.1371/journal.pbio.2003533

**Published:** 2017-12-28

**Authors:** Kristofer Wollein Waldetoft, Sam P. Brown

**Affiliations:** School of Biological Sciences, Georgia Institute of Technology, Atlanta, Georgia, United States of America; Pennsylvania State University, United States of America

## Abstract

Alternative therapeutics for infectious diseases is a top priority, but what infections should be the primary targets? At present there is a focus on therapies for severe infections, for which effective treatment is most needed, but these infections are hard to manage, and progress has been limited. Here, we explore a different approach. Applying an evolutionary perspective to a review of antibiotic prescription studies, we identify infections that likely make a large contribution to resistance evolution across multiple taxa but are clinically mild and thus present easier targets for therapeutics development. Alternative therapeutics for these infections, we argue, would save lives indirectly by preserving the high efficacy of existing antibiotics for the patients who need them the most.

## Introduction

The evolution of antimicrobial resistance is among the greatest challenges to public health in our time [1], and the development of novel therapeutics to replace traditional antibiotics will likely play a key role in addressing this issue [2]. The state of the pipeline of alternative therapeutics was recently assessed in a report commissioned by the Wellcome Trust [2]. Focusing on "therapies that could be developed to treat systemic/invasive rather than superficial infections" [2], the authors assessed the potential of the major current lines of research in the area, including bacteriophages, phage lytic enzymes, probiotics, and antimicrobial peptides [2]. At present, however, candidate therapies are a far cry from serious contenders to antibiotics in the treatment of severe infections, and the authors of the report conclude that, rather than being true alternatives, these therapeutics may be "used as adjuncts to antibiotics because their activities may not provide sufficient therapeutic benefit on their own" [2].

In addition to the efforts made in the biomedical field, there is growing interest among evolutionary biologists in developing drugs that are more robust to the evolution of resistance. This can be done, for example, by targeting traits that are shared among bacterial cells, such as quorum sensing or siderophores, and thus taking advantage of evolutionary conflicts between groups and individuals [3,4]. While these strategies promise to be less prone to resistance evolution, they only inhibit bacterial proliferation to a limited extent and are thus unlikely to be as effective treatments as classic antibiotics that efficiently kill the pathogen. And one would be hard pressed to rely on such a drug as the sole antibacterial in treating a severe invasive infection.

Thus, novel types of therapeutics are being developed, but they share the problem that, plainly put, they are not good enough for the treatment of severe infections. In this paper, we take one step back and address the fundamental question of what function alternative therapeutics for infectious diseases should serve, and we outline a way forward through a focus on common mild infections.

## The dual roles of alternatives to antibiotics

There are at least 2 ways in which alternative therapeutics can help alleviate the resistance crisis. First, they can be used to manage infections that are no longer treatable with antibiotics because the bacteria are resistant. This role entails a focus on severe infections, for which effective treatment is most needed, and therefore puts high demands on efficacy. It is a goal promoted by WHO [5], which we are currently failing to achieve [2].

Second, alternative therapeutics can substitute for antibiotics in order to decrease antibiotic consumption and thus relax selection for resistance. In this case, the focus should rather be on infections that make a large contribution to resistance evolution and are realistic targets for alternative therapeutics.

## For which infections are alternative therapeutics most feasible?

The reasons for the current lack of success (see above) are likely complex, but we think an important aspect is the focus on infections that are severe (e.g., sepsis) and therefore hard to treat. By turning our attention to infections that are mild and self-limiting [6] (e.g., strep throat), we can set the bar lower and thus plausibly increase the success rate.

Mild infections might also be more readily treated with novel therapeutic options, including antivirulence drugs, quorum quenchers, and other therapeutics that may be less effective treatments but can be made more robust to resistance evolution than traditional antibiotics [3]. Furthermore, because mild infections leave more time for diagnostics than do severe infections that need to be treated without delay [7], they lend themselves better to therapeutics that have a narrow spectrum and thereby a smaller negative impact on the commensal biota.

## In mild infections, alternative drugs can be better for the patient

The best option for the patient is that which strikes the best balance between treatment benefit and side effects. For antibiotics, the latter include an increased risk of future antibiotic resistance [8] and antibiotic-associated diarrhoea [9]. Because alternative therapeutics are not antibiotics and are often narrow spectrum (e.g., phages, lysins, specific virulence factor inhibitors), it is plausible that these side effects would be less pronounced. (The potential for other types of side effects is hard to assess for alternatives in general.) Alternative therapeutics can therefore be better for the patient, given that the infection is sufficiently mild that the difference in treatment efficacy does not outweigh the difference in these side effects.

## Are mild infections important for the evolution of resistance?

It is uncontroversial that the evolution of antibiotic resistance is driven by antibiotic use, but the quantitative contribution of mild infections to clinically important resistance is incompletely known. Here, we use a thought experiment that is grounded in evolutionary theory and in line with empirical data to discuss the contribution of antibiotic consumption in the community to medically relevant resistance.

Consider the following scenario. An individual harbours *Escherichia coli* strains with and without genes encoding β-lactamases in an asymptomatic carriage state. The individual then contracts streptococcal pharyngitis, consults the general practitioner, and is prescribed amoxicillin. *E*. *coli* β-lactamases confer resistance to amoxicillin [10], and under treatment this gives the β-lactamase–producing strain a competitive advantage over the nonproducing strain. The β-lactamase–producing strain thus increases in abundance in the patient's microbiota, temporarily or more permanently. This, in turn, increases the probability that it will transmit to other individuals or spread to an anatomical site where it causes disease, thus contributing to the resistance problem in the individual patient as well as society.

Consistent with this scenario, a 1-week course of amoxicillin given to healthy volunteers resulted in an increase in *E*. *coli* positive for a β-lactamase gene in faeces [11], and a single dose of oral ampicillin administered to patients with gonorrhoea was followed by an increase in faecal *E*. *coli* resistant to this drug [12]. In addition, previous use of penicillin is a risk factor for extended spectrum β-lactamase (ESBL) and multidrug-resistant enterobacteriaceae in urinary tract infections in the community [13,14]. The relevance of this for severe infection is suggested by the finding that carriage of ESBL positive *E*. *coli* is a risk factor for bacteraemia with these organisms [15].

This thought experiment illustrates 2 general points. First, systemic antibiotic treatment selects for resistance throughout the patient's microbiota, not only in the pathogen it is aimed to target. Thus, when weighing the benefits of treatment against the problem of selecting for resistance, one needs to look beyond the infection at hand and take the whole microbiota into account.

Second, the use of a single antibiotic can contribute to the increase in resistance to a whole range of drugs if resistance determinants co-occur in the same bacterial strains, which is often the case [10]. β-lactamases are cases in point, especially ESBLs, as they confer resistance to several antibiotics in the β-lactam group (e.g., penicillins and cephalosporins [16]). But also, genes that encode unrelated resistance mechanisms may occur together on the same mobile genetic elements or otherwise in the same bacteria. If a strain is resistant to several antibiotics, it will be favoured by selection imposed by any of those drugs, and when the strain increases in frequency due to this selection, so do all resistance determinants that it encodes. Multiresistant strains thus allow different resistance determinants to 'hitchhike' [17] with each other. In terms of our thought experiment with β-lactamase producing *E*. *coli*, the relevance of this effect, even for mechanistically unrelated resistance determinants, is suggested by the co-occurrence of resistance to third generation cephalosporins and fluoroquinolones, and the finding that previous use of quinolones is a risk factor for ESBL in community-acquired urinary tract infections [10,13,18]. The problem of multiresistance may be further exacerbated by the fact that multiresistant strains are favoured under a larger number of treatment regimens, and thereby in a larger fraction of patients, as compared to strains that are resistant to a single type of antibiotic.

More generally, systematic reviews and meta-analyses have found associations between antibiotic consumption and resistance in the community [19] as well as between antibiotic prescription in primary care and resistance in the individual patient [8], and there is an epidemiological association between antibiotic prescription in the community and resistance in invasive infection [20–22]. Whilst these studies leave much open, they do suggest a role for antibiotic consumption in the community in the spread of medically important resistance. This is unsurprising, because the community dominates antibiotic use. For example, in the European Union (2011) a median of 19.5 defined daily doses (DDDs) were prescribed per 1,000 inhabitants per day in the community, as compared to 1.8 DDDs per 1,000 inhabitants per day in the hospital sector [23].

In summary, the evolutionary consequences of antibiotic treatment go beyond the antibiotic used and the pathogen at which it is aimed, and it is plausible that prescription in the community makes a relevant contribution to resistance. Next, we turn to the indications that drive this antibiotic use.

## Which infections contribute most to antibiotic consumption in the community?

To address this question, we review the published literature on antibiotic prescription in the community (see S1 Text for details).

Antibiotic prescription in the community has been studied in a number of countries [24–44], and whilst differences in diagnostic classifications among studies prevent detailed comparisons, a general pattern is clear: a large proportion of prescriptions are due to infections that in previously healthy individuals are typically mild and self-limiting. Many cases pertain to the upper respiratory tract and adjacent structures. Indeed, in the United States, sinusitis, otitis media, and pharyngitis rank first, second, and third, respectively, and together contribute almost 30% of the prescriptions [24].

Overall, pharyngitis/tonsillitis and urinary tract infection are among the top 4 indications in more than half of the studies, and they contribute a median of 8.5% and 9.3% of the prescriptions, respectively. There is variation among countries, however, and, for example, in Sweden these 2 diagnoses account for 19% and 20%, respectively.

**Fig 1:**
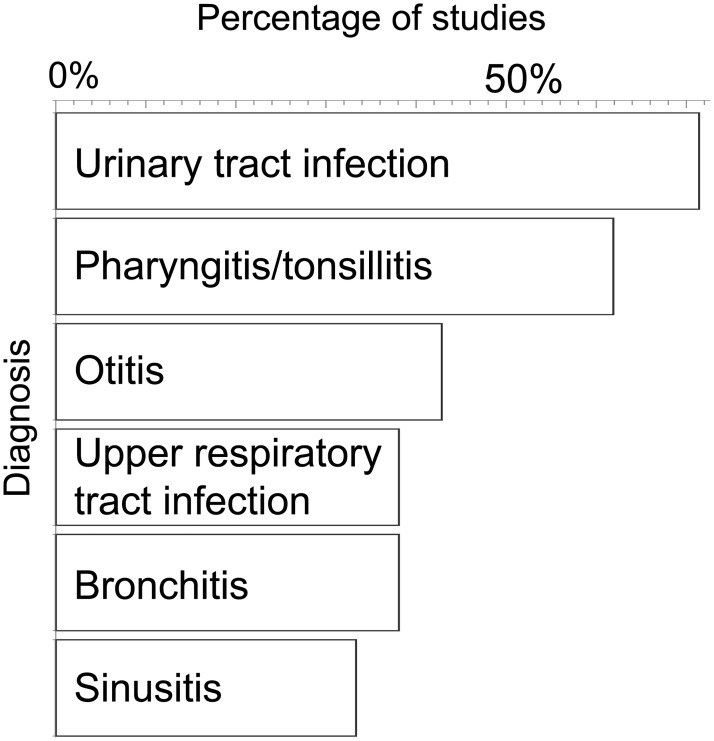
Mild infections make a large contribution to antibiotic prescription. The literature on antibiotic prescription in the community was reviewed, and the top 4 indications in each country studied were extracted. The 6 diagnoses that were among the top 4 in the largest proportion of studies are presented above, with bars representing the proportion of studies (countries, *n* = 21) in which the diagnosis was among the top 4 (see S1 Text for details).

There is thus scope for considerably reducing antibiotic use by developing alternative therapeutics for infections that are relatively mild. For the fraction of these infections that are viral, it is possible to decrease antibiotic prescription even in the absence of alternatives. A summary of the findings is given in Fig 1, and details are given in S1 Text.

## Streptococcal pharyngotonsillitis as a candidate for alternative therapeutics

In this section, we discuss possible alternative treatments for pharyngotonsillitis. We discuss this condition, specifically, because it makes an important contribution to antibiotic consumption (see above), but the alternative treatment strategies available and the types of issues one needs to consider are relevant to other conditions as well.

Bacterial pharyngotonsillitis is almost always of streptococcal aetiology, with group A streptococci (GAS) accounting for the majority of cases [45]. For GAS pharyngitis, penicillins are the treatment of choice [46], and despite extensive penicillin use, GAS are invariably sensitive. Penicillin treatment of GAS infections may thus seem unproblematic from a resistance perspective. However, because these infections make a large contribution to antibiotic use, they may affect the evolution of resistance in other bacteria, in accordance with the thought experiment and discussion above.

In assessing the suitability of streptococcal pharyngitis for alternative therapeutics, it is key to consider the reasons why this condition is currently treated with antibiotics. In their guidelines for the management of GAS pharyngitis, the Infectious Diseases Society of America (IDSA) invokes the following reasons for treatment: prevention of acute rheumatic fever (ARF), prevention of suppurative complications, improvement of clinical signs and symptoms, reduced contagiousness/transmission, and accelerated resumption of normal activities [46]. To this may be added the prevention of Lemièrre's syndrome due to, e.g., *Fusobacterium necrophorum* pharyngitis, which may be misdiagnosed as streptococcal. The latter risk should, however, be small, given adequate diagnostic procedures.

The effect of antibiotic treatment in sore throat, including streptococcal, has been assessed in a systematic review from the Cochrane Library [47]. The authors conclude that "the absolute benefits are modest" [47] but point to the problem of context dependence. The case of ARF serves to illustrate this problem. Antibiotic treatment decreases the risk of ARF to approximately one-fourth [47]. However, the incidence of ARF varies greatly among populations [48], and the absolute risk of this sequela has to be locally assessed. For example, in Sweden, current treatment guidelines state that the risk of ARF is too low to motivate the use of antibiotics [49]. (Antibiotic treatment is still recommended on other grounds.) The issue of context dependence applies to suppurative complications, as well [47]. We therefore conclude that an alternative therapeutic for streptococcal pharyngitis should ideally address all of the reasons for treatment listed in the IDSA guidelines, but the minimum acceptable level of effectiveness in preventing sequelae will differ among populations. The reduction of symptoms and shortening of the infection period are, on the other hand, relevant to all populations. In the following, we discuss alternative approaches that we think have potential.

Asymptomatic carriage of GAS is common and does not normally warrant antibiotic treatment, as the risks for complications and transmission are considered low [46]. One potential strategy would therefore be to develop antivirulence drugs that turn symptomatic infection into asymptomatic carriage, decrease the symptoms and risks associated with infection, or help the immune system to clear the bacteria. Specific factors of interest include the superantigens, such as the streptococcal pyrogenic exotoxin A, which induce inflammation and may contribute to both symptoms and bacterial burden in pharyngitis [50,51], and streptokinase, which has been implicated in the evasion of host defence in the microvasculature as well as saliva [52,53]. In addition, both the superantigens and streptokinase are likely to be bacterial public goods and thus lend themselves to more evolutionarily robust therapeutics [3], although the fact that different strains have different superantigen profiles suggests the possibility of strain replacement in response to interventions. Although the feasibility of this approach is hard to assess, the fact that the virulence mechanisms of GAS have been extensively studied at the molecular level speaks in its favour.

Another approach would be to use bacteriophages. As compared to the antivirulence approach, this would have the benefit of killing the bacteria and thus make it more similar to current treatment. A recent study in the Russian military reported promising results using phages as prophylaxis for respiratory tract infections, including tonsillitis [54]. We would interpret this study cautiously, however, because the published full text is incomplete, with some pages omitted.

The mechanisms by which phages kill streptococci are known, and work on sequestering these mechanisms for therapeutic purposes has made substantial progress. Thus, phage lytic enzymes have been shown to be effective against GAS in mouse models of invasive infection [55] as well as oral colonization [56].

The therapeutic avenues discussed above have the potential to address all of the reasons for current antibiotic treatment of GAS pharyngotonsillitis, as listed in the IDSA guidelines. In addition, the therapeutics would be more specific to GAS than are current antibiotics. In the case of antivirulence drugs, this is due to the fact that specific molecular mechanisms of virulence would be targeted, while the narrow host range of phages has a similar effect for therapy with phages and their lytic enzymes. These potential therapeutics should therefore be less disruptive to the patient's microbiota and impose less selection on other bacteria, as compared to traditional antibiotics.

## Discussion and conclusion

Here, we have made 2 interconnected points. First, infections that are relatively mild make a large contribution to antibiotic prescription, and it is plausible that this translates into considerable selection pressure for antibiotic resistance.

Second, by turning our attention to infections that make a large contribution to resistance evolution but are clinically mild, we may increase the success rate of alternative therapeutics development and decrease the selection pressure for resistance to current antibiotics. We have focussed on the contribution to bulk antibiotic consumption, but more subtle aspects are also relevant. For example, different groups of patients (e.g., age groups) differ in the incidence of specific infections as well as in the bacteria they carry asymptomatically and their co-occurring medical conditions and histories, and this may affect the type of resistance being prevented.

In conclusion, together with other strategies, such as shorter courses [8] and the use of more narrow spectrum antibiotics [12], where applicable, alternative therapeutics for mild infections would decrease the selection pressure for antibiotic resistance and thus help retain the efficacy of current antibiotics, so that we can keep using them to treat the severe infections for which we fail to develop novel drugs.

## Supporting information

S1 Text The literature on antibiotic prescription in the community was reviewed. S1 Text provides the search strategy, the method for study selection, the studies included, and the data underlying the summary of the findings given in the main text and Fig 1.
(PDF)

## Abbreviations

ARF: acute rheumatic fever
DDD: defined daily dose
ESBL: extended spectrum β-lactamase
GAS: group A streptococci
IDSA: Infectious Diseases Society of America
