# Supplementary material for: Alternative therapeutics for self-limiting infections—An indirect approach to the antibiotic resistance challenge
Source: PLoS Biol. 2017 Dec 28;15(12):e2003533. doi: 10.1371/journal.pbio.2003533 (PMC5746204; doi:10.1371/journal.pbio.2003533)
Supplement: S1 Text — provides the search strategy, the method for study selection, the studies included, and the data underlying the summary of the findings given in the main text and Fig 1. (PDF) [file pbio.2003533.s001.pdf]

Supplement

This supplement provides a summary of the published literature on antibiotic prescription in primary care / ambulatory care / general practice / for outpatients.

Background

Goal

The goal was to determine the top four indications for antibiotic prescription in primary care / ambulatory care / general practice / for outpatients, and the proportion of prescriptions that are due to each of those indications in each of the countries / areas studied. The cutoff at four diagnoses was applied in order to keep the number of diagnoses constant across studies.

Search strategy

The PubMed database was searched 2017-01-02 with the search term “prescri*[tiab] AND (antibiotic*[ti] OR antibacterial[ti]) AND (“primary care”[ti] OR “ambulatory care”[ti] OR outpatient[ti] OR outpatients[ti] OR “general practice”[ti] OR “general practitioner”[ti] OR “general practitioners”[ti] OR community[ti])”, yielding 818 hits.

Selection of studies

The entries of the PubMed result list were reviewed to assess whether the paper was likely to include data on antibiotic prescription by indication in primary care / ambulatory care / general practice / for outpatients. Studies that only considered specific groups of diagnoses (e.g. respiratory tract infections), specific patient categories (e.g. paediatric), or specific categories of prescribers within the category of health care studied (e.g. physicians’ assistants) were excluded. Studies of general practitioners and general practitioners receiving doctors in training were included. One questionnaire based study (Lam, Ho, Lam, Choi, & Yung, 2009) was excluded due to very low response rates. For each country only the most recent study was included, with the exception that for Sweden a nation wide study (Nord, Engström, & Mölstad, 2013) was included instead of a more recent study of a single county (Neumark, Brudin, & Mölstad, 2015). Publications in Danish, English, French, German, Norwegian, Spanish, and Swedish were considered. First, the titles were assessed. If the title was selected, the abstract was assessed, and if the abstract was selected, the figures and tables were assessed.

Countries and studies included

USA (Fleming-Dutra et al., 2016).
Malaysia (Ab Rahman, Cheong, & Sivasampu, 2016).
Indonesia (Andrajati, Tilaqza, & Supardi, 2015).
Lebanon (Saleh et al., 2015).
France (Etienne & Pulcini, 2015).
Spain (Fernández-Urrusuno et al., 2014).
The Netherlands (van den Broek d’Obrenan, Verheij, Numans, & van der Velden, 2014).
Sweden (Nord et al., 2013).
Latvia (Dumpis, Dimiņa, Akermanis, Tirāns, & Veide, 2013).

Ireland (Murphy, Bradley, & Byrne, 2012).
India (Pathak et al., 2011).
United Kingdom (Petersen & Hayward, 2007).
Turkey (Avci et al., 2006).
Australia (Pan, Henderson, & Britt, 2006).
Canada (Jelinski, Parfrey, & Hutchinson, 2005).
Finland (Rautakorpi et al., 2001).
Taiwan (Chang, Shiu, & Chen, 2001).
Schweiz (Stürchler, Vuille, Zemp, Tschud, & Zimmerli, 2000).
Norway (Straand, Rokstad, & Sandvik, 1998).
Denmark (Gade-Kristensen, Knudsen, Møller, Sidenius, & Osterbye, 1991).
Nigeria (Obaseiki-Ebor, Akerele, & Ebea, 1987).

Results

Table S1

The top four indications for antibiotic prescription in primary care / ambulatory care / general practice / for outpatients by country, year of publication, and diagnosis. The numbers are percent of the total number of prescriptions in the study. Diagnosis names are kept close to the original.

USA 2016	Sinusitis	11.0672
	Suppurative otitis media	9.28854
	Pharyngitis	8.49802
	Urinary tract infection	6.917
Malaysia 2016	Acute upper respiratory tract infection (R74)	49.2
	Acute tonsillitis (R76)	6.
	Cystitis/urinary infection (U71)	4.5
	Gastroenteritis (D73)	3.9
Indonesia 2015	Acute pharyngitis	40.2
	Non-specific acute respiratory infection	25.4
	Acute tonsillitis	5.3
	Common cold and Diarrhoea, respectively	3.7
Lebanon 2015	Respiratory infection	41
	Urinary tract infection	14
	Gastrointestinal (diarrhoea)	11
	Oral infection	10
France 2015	Acute pneumonia	18.9655
	Cystitis (UTI)	15.0862
	Pharyngitis/Tonsillitis	11.6379
	Acute bronchitis	6.89655
Spain 2014	Pharyngotonsillitis	18.4834
	Urinary tract infection	8.21485
	Skin infection	6.47709
	Otitis	6.08215
The Netherlands 2014	Cystitis (U71)	18.8674
	Acute upper respiratory tract infection (R74)	6.28462
	Acute otitis media (H71)	5.85137
	Sinusitis (R75)	5.4188

Sweden 2013	Cystitis	20
	Tonsillitis and Pharyngitis	19
	Otitis Media	15
	Sinusitis	8
Latvia 2013	Pharyngitis	29.8
	Acute bronchitis	25.3
	Rhinosinusitis	10.2
	Pneumonia	7.8
Ireland 2012	Skin infection	10.21
	Tonsillitis	8.688
	Urinary tract disorders	8.63
	Otitis media	6.16378
India 2011	Vaginal discharge	26.3302
	Upper respiratory tract infection	19.4758
	Urinary tract infection	16.6604
	Skin and soft tissue infection	14.2363
United Kingdom 2007	Upper respiratory tract infection	14.5
	Lower respiratory tract infection	13.
	Sore throat	11.4
	Urinary tract infection	8.3
Turkey 2006	Upper respiratory tract infection	45.4
	Urinary tract infection	11.4
	Dental infection	5.5
	Lower respiratory tract infection	4.2
Australia 2006	Upper respiratory tract infection	16.4
	Acute bronchitis/bronchiolitis	14.5
	Urinary tract infection	8.3
	Acute otitis media	8.
Canada 2005	Pharyngitis	23.7435
	Otitis media	16.9844
	Bronchitis	12.6516
	Sinusitis	12.305
Finland 2001	Otitis media	28.9722
	Sinusitis	20.6113
	Throat infection	11.1033
	Acute bronchitis	10.556
Taiwan 2001	Common cold	29.4
	Acute bronchitis	6.3
	COPD or Asthma	4.1
	Skin and subcutaneous	4.1
Schweiz 2000	Cystitis	19.9
	Acute sinusitis	14.1
	Acute bronchitis	11.5
	Tonsillopharyngitis	9.2
Norway 1998	Urinary tract infection	24
	Acute bronchitis	13
	Ear infection	9
	Acute tonsillitis	8.2
Denmark 1991	Upper respiratory tract infection	39
	Lower respiratory tract infection	18
	Urogenital infection	18
	Skin infection	7

Nigeria 1987	Soft tissue infection	37.9
	Upper respiratory tract infection	10.6
	Malaria	8.4
	Gastroenteritis/vomiting	7.4

Proportion of studies in which a particular diagnosis is among the top 4

This is the basis for figure 1.

Urinary tract infection (UTI) includes diagnosis names containing “urinary tract infection”, “cystitis”, “urinary tract disorders” (one country), or “urogenital infection” (one country).

Pharyngitis/tonsillitis (Pharyng.) includes diagnosis names containing “pharyngitis”, “tonsillitis” or their combination, “sore throat”, or “throat infection”. When both pharyngitis and tonsillitis were among the top four indications in a given study (only one study), they were counted as one occurrence.

Otitis includes diagnosis names containing “otitis” (typically “otitis media”) and “ear infection”.

Upper respiratory tract infection (URTI) includes diagnosis names containing “upper respiratory tract infection”.

Bronchitis (Bronch.) includes diagnosis names containing “bronchitis” (typically “acute bronchitis”).

Sinusitis includes diagnosis names containing “sinusitis” or “rhinosinusitis”.

Median percentage of prescriptions attributable to pharyngitis/tonsillitis and urinary tract infection

Medians were determined for those diagnoses that are among the top 4 in more than half of the studies. They were calculated based the data for all diagnoses in each study, not only the top 4.

Pharyngitis/tonsillitis

This includes diagnosis names containing “pharyngitis”, “tonsillitis” or their combination, “sore throat”, or “throat infection”. When both pharyngitis and tonsillitis were among the top four indications, their contributions were summed.

```
8.5
```

Urinary tract infection (UTI)

This includes diagnosis names containing “urinary tract infection”, “cystitis”, “urinary tract disorders” (one country), or “urogenital infection” (one country).

```
9.3
```

References

Ab Rahman, N., Cheong, L. T., & Sivasampu, S. (2016). PATTERN-Antibiotic prescribing in public and private practice: a cross-sectional study in primary care clinics in Malaysia. BMC Infectious

Diseases, 16(1), 208. http://doi.org/10.1186/s12879-016-1530-2
Andrajati, R., Tilaqza, A., & Supardi, S. (2015). Factors related to rational antibiotic prescriptions in community health centers in Depok City, Indonesia. Journal of Infection and Public Health, 10(1), 41-48. http://doi.org/10.1016/j.jiph.2016.01.012
Avci, I. Y., Kilic, S., Acikel, C. H., Ucar, M., Hasde, M., Eyigun, C. P., … Cetiner, S. (2006). Outpatient prescription of oral antibiotics in a training hospital in Turkey: Trends in the last decade. Journal of Infection, 52(1), 9-14. http://doi.org/10.1016/j.jinf.2005.07.007
Chang, S.-C., Shiu, M.-N., & Chen, T.-J. (2001). Antibiotic usage in primary care units in Taiwan after the institution of national health insurance. Diagnostic Microbiology and Infectious Disease, 40(3), 137-143. Retrieved from http://www.embase.com/search/results?subaction=viewrecord&from=export&id=L32735553%5Cnhttp://dx.doi.org/10.1016/S0732-8893(01)00256-5%5Cnhttp://sfx.library.uu.nl/utrecht?sid=EMBASE&issn=07328893&id=doi:10.1016%2FS0732-8893%2801%2900256-5&atitle=Antibiotic
Dumpis, U., Dimiņa, E., Akermanis, M., Tirāns, E., & Veide, S. (2013). Assessment of antibiotic prescribing in Latvian general practitioners. BMC Family Practice, 14, 9. http://doi.org/10.1186/1471-2296-14-9
Etienne, C., & Pulcini, C. (2015). Évaluation prospective des prescriptions antibiotiques d'un échantillon de médecins généralistes français. Presse Medicale, 44(3), e59-e66. http://doi.org/10.1016/j.lpm.2014.07.022
Fernández-Urrusuno, R., Flores-Dorado, M., Vilches-Arenas, A., Serrano-Martino, C., Corral-Baena, S., & Montero-Balosa, M. C. (2014). Adecuación de la prescripción de antibióticos en un área de atención primaria: estudio descriptivo transversal. Enfermedades Infecciosas Y Microbiologia Clinica, 32(5), 285-292. http://doi.org/10.1016/j.eimc.2013.05.004
Fleming-Dutra, K. E., Hersh, A. L., Shapiro, D. J., Bartoces, M., Enns, E. A., File, T. M., … E, H. (2016). Prevalence of Inappropriate Antibiotic Prescriptions Among US Ambulatory Care Visits, 2010-2011. Jama, 315(17), 1864. http://doi.org/10.1001/jama.2016.4151
Gade-Kristensen, A., Knudsen, E., Møller, B., Sidenius, J., & Osterbye, P. (1991). Treatment of infectious diseases with antibacterial drugs in general practice. Ugeskr Laeger, 153(3), 176-81.
Jelinski, S., Parfrey, P., & Hutchinson, J. (2005). Antibiotic utilisation in community practices: Guideline concurrence and prescription necessity. Pharmacoepidemiology and Drug Safety, 14(5), 319-326. http://doi.org/10.1002/pds.1007
Lam, T. P., Ho, P. L., Lam, K. F., Choi, K., & Yung, R. (2009). Use of antibiotics by primary care doctors in Hong Kong. Asia Pacific Family Medicine, 8(1), 5. http://doi.org/10.1186/1447-056X-8-5
Murphy, M., Bradley, C. P., & Byrne, S. (2012). Antibiotic prescribing in primary care, adherence to guidelines and unnecessary prescribing--an Irish perspective. BMC Family Practice, 13, 43. http://doi.org/10.1186/1471-2296-13-43
Neumark, T., Brudin, L., & Mölstad, S. (2015). Antibiotic prescribing in primary care by international medical graduates and graduates from Swedish medical schools. Family Practice, 32(3), 343-347. http://doi.org/10.1093/fampra/cmv001
Nord, M., Engström, S., & Mölstad, S. (2013). Mycket varierande förskrivning av antibiotika i primärvården. Läkartidningen, 110, 1-3.
Obaseiki-Ebor, E. E., Akerele, J. O., & Ebea, P. O. (1987). A survey of antibiotic outpatient prescribing and antibiotic self-medication. Journal of Antimicrobial Chemotherapy, 20(5), 759-763. http://doi.org/10.1093/jac/20.5.759
Pan, Y., Henderson, J., & Britt, H. (2006). Antibiotic prescribing in Australian general practice: How has it changed from 1990-91 to 2002-03? Respiratory Medicine, 100(11), 2004-2011. http://doi.org/10.1016/j.rmed.2006.02.015
Pathak, A., Mahadik, K., Dhaneria, S. P., Sharma, A., Eriksson, B., & Lundborg, C. S. (2011). Antibiotic prescribing in outpatients: Hospital and seasonal variations in Ujjain, India. Scandinavian Journal of Infectious Diseases, 43(6-7), 479-88. http://doi.org/10.3109/00365548.2011.554854
Petersen, I., & Hayward, A. C. (2007). Antibacterial prescribing in primary care. Journal of Antimicrobial Chemotherapy, 60(SUPPL. 1). http://doi.org/10.1093/jac/dkm156

Rautakorpi, U.-M., Klaukka, T., Honkanen, P., Mäkelä, M., Nikkarinen, T., Palva, E., … Group), (MIKSTRA collaborative study. (2001). Antibiotic Use by Indication: A Basis for Active Antibiotic Policy in the Community. Scandinavian Journal of Infectious Diseases, 33(12), 920-926. http://-doi.org/10.1080/00365540110077056
Saleh, N., Awada, S., Awwad, R., Jibai, S., Arfoul, C., Zaiter, L., … Salameh, P. (2015). Evaluation of antibiotic prescription in the Lebanese community: a pilot study. Infection Ecology & Epidemiology, 5, 27094. http://doi.org/10.3402/iee.v5.27094
Straand, J., Rokstad, K., & Sandvik, H. (1998). Prescribing systemic antibiotics in general practice. A report from the Møre & Romsdal Prescription Study. Scand J Prim Health Care, 16(2), 121-7.
Stürchler, M., Vuille, P., Zemp, E., Tschud, i P., & Zimmerli, W. (2000). Diagnosis and antibiotic therapy of infections in outpatients. Schweiz Med Wochenschr., 130(41), 1437-46.
van den Broek d’Obrenan, J., Verheij, T. J. M., Numans, M. E., & van der Velden, A. W. (2014). Antibiotic use in Dutch primary care: Relation between diagnosis, consultation and treatment. Journal of Antimicrobial Chemotherapy, 69(6), 1701-1707. http://doi.org/10.1093/jac/dku005
